# Development of a food atlas for Sri Lankan adults

**DOI:** 10.1186/s40795-017-0160-4

**Published:** 2017-05-25

**Authors:** Ranil Jayawardena, Manoja P. Herath

**Affiliations:** 1 Department of Physiology, Faculty of Medicine, University of Colombo, Colombo, Sri Lanka; 2 Institute of Health and Biomedical Innovation, Queensland University of Technology, Brisbane, QLD Australia; 3 Diabetes Research Unit, Department of Clinical Medicine, Faculty of Medicine, University of Colombo, Colombo, Sri Lanka

**Keywords:** Food atlas, Sri Lanka, Adults, Food photography

## Abstract

**Background:**

Obtaining reliable food portion size estimations in dietary surveys found to be a difficult task. A food atlas is a set of photograph series depicting different amounts of a particular food, usually bound together in a single volume that can be used to describe portion sizes. By this paper we aim to explain the methods used in developing a photographic food atlas for Sri Lankan adults and to discuss its usage, advantages and limitations.

**Methods:**

Foods that are commonly consumed in Sri Lanka were recognized by a local nutritional survey, market survey and interviewing nutrition experts. In obtaining food items, certain dishes were prepared according to a standard recipe book while other items were purchased from recognized restaurants and the local market. White color crockery or/with blue color board was used to present the food items and they were photographed by a professional photographer employing a camera setup recommended for food photography. Three types of photographs have been used to illustrate the food items; serial, range and guide. Mainly the serial photographs were taken using two cameras: one fixed at an angle of 5° to capture aerial views, and the other placed at an angle of 45° to represent the view of a person of average height, sitting at a table, looking at a plate on the table in front of him. The liquid portion series were photographed at an angle of 90° to get life-size images. The range and guide photographs were taken free hand so that all the items could be captured in the best way possible.

**Results:**

A total of 125 foods that are commonly consumed by Sri Lankan adults were selected to be depicted in the atlas; serial photographs of increasing portion sizes (*n* = 88 foods); range photographs depicting a range of sizes/varieties of a particular food, (*n* = 11), and guide photographs which represent the brands/types of a certain food item/category available in the market (*n* = 26).

**Conclusion:**

The food atlas will be a valuable resource for dietary surveys in Sri Lanka as well as in other South Asian countries where similar foods are consumed.

**Electronic supplementary material:**

The online version of this article (doi:10.1186/s40795-017-0160-4) contains supplementary material, which is available to authorized users.

## Introduction

Nutrition transition that is affected by a wide range of socioeconomic and demographic shifts has resulted in rapid changes in the diets of most regions of the world. These involve increases in refined carbohydrate intakes, replacement of high fiber foods by processed versions, and increases in intakes of animal and partially hydrogenated fats [1]. Studies have identified that an unhealthy diet is a major risk factor for the global burden of chronic non-communicable diseases (NCDs) including obesity, diabetes, hypertension, stroke, hyperlipidaemia, coronary heart disease, and cancer [2, 3]. A total of 38 million deaths occurred globally due to NCDs during 2012, and almost three quarters of these deaths (28 million) occurred in low and middle-income countries [4].

Sri Lanka, as a low middle income country, has also experienced this so called nutrition transition in recent decades which has lead the country to suffer from the double burden of under- and over-nutrition [5]. The trends in risk factors such as increased consumption of energy-dense, nutrient-poor foods that have high amounts of fat, sugar and salt, for increased NCD-related morbidity and mortality, manifest an upward direction [6]. It has been reported that diet-related chronic diseases currently account for an estimated 18.3% of total mortality and 16.7% of hospital expenditure in Sri Lanka [3]. According to recent studies, the corresponding percentages of Sri Lankan adults in the overweight, obese and centrally obese categories were 25.2, 9.2 and 26.2% [7] and the age-adjusted prevalence for metabolic syndrome was 24.3% [8] while 23.7% adults were suffering from hypertension [9], with one in every five adults aged above 20 years having either diabetes or pre-diabetes [10]. On the other hand, a high prevalence of malnutrition among hospitalized patients has been reported recently [11]. In addition, the prevalence of stunting, underweight, wasting and anaemia among children were 7.1, 16.9, 21.2 and 7.4%, respectively [12].The prevalence of under nutrition among the institutionalized elderly living in Sri Lanka was 30% [13]. Moreover, iron deficiency anemia among pregnant women is reaching an epidemic proportion [14].

Prevention of diet-related chronic diseases at a population level is important [15] however, nutrition intervention should be implemented at individual level; not only because an individual’s age and sex will make a difference in food consumption, but also because each person will differ in height and weight and will have diverse dietary habits. It is well known that prior to prevention, the problem must be well identified; in this case, the habitual dietary intake of the individuals should be measured. The data that is thereby obtained will be useful in identifying nutritional status, monitoring dietary practices and studying the relationships between diet and diseases [16]. The methods used to quantify food intakes of individuals can be divided into two broad categories: (i) those in which subjects are asked to weigh the foods before and after eating (ii) those in which subjects are asked to recall of food quantities they have consumed [17]. The most accurate method for measuring food intake is weighing foods but it is time consuming, expensive [18], and as it imposes a large burden on participants [19], there is high tendency of subjects under-reporting the intakes [17]. Moreover, it cannot be used in epidemiological surveys involving large numbers of people. The second type of dietary assessment tools, for example 24-h dietary recall (24HR), dietary record (DR), dietary history (DH), and frequency questionnaires (FFQ), comprise portion size estimation with the help of a trained interviewer or by self-reporting. Therefore, the subject has to recall the amount eaten accurately [20]. However, obtaining reliable food portion size estimates from subjects is found to be a difficult task, unless they are assisted with visual aids such as a set of photographs depicting different amounts of a particular food [21].

A Food atlas is a set of photograph series depicting different amounts of a particular food, usually bound together in a single volume [22]. The photographs represent the range of portion sizes consumed in reality by the particular population and the subjects have to select the photograph that reflects either their usual portion size or the actual portion size, according to the dietary survey method used [18]. The literature has emphasized the importance of food photographs, in improving the accuracy of food quantification [23]. In earlier dietary studies in Sri Lanka, portion sizes were estimated either by recalling utensils used in general or demonstrating standard spoons, cups and plates, in addition to food photographs [16]. However, the existing pictures did not cover the commonly consumed foods in appropriate portion sizes. For instance, a WHO STEP survey conducted in 2004, states that the researchers had difficulty in assessing the portion/serving size of vegetables [6]. Therefore, a food atlas was identified as an essential but absent tool for the quantification of food portion sizes in dietary surveys in Sri Lanka. The atlas can be incorporated into any method in which food quantities are estimated. Anyway, it will be most advantageous in a diet history interview or 24-h recall [24].

The aim of this paper is to explain the methods used to develop a photographic food atlas of commonly consumed traditional and non-traditional foods in Sri Lanka and to discuss its usage, advantages and limitations.

## Methods

### Literature review

A number of food atlases: Australia, Malaysia, United Arab Emirates (UAE), United Kingdom (UK) and United States of America (USA)were studied with reference to what food items have been included, how many portion sizes of each food item have been incorporated, what types of crockery have been used to place the foods, what types of cutlery have been used as reference measurements and how food items that cannot be measured as portions, but comprise various sizes have been interpreted. Additionally, methodology articles on food atlases were reviewed [22, 25].

### Selecting food items

Foods that are commonly consumed in Sri Lanka were recognized by a nutritional survey data and by data from the development phase of the food frequency questionnaire (FFQ) for Sri Lankan adults [26]. Almost all the items in the FFQ have been included in the atlas apart from a few items with more or less similar consumption patterns and nutrients for which separate photographs were not required, because their portion sizes can be estimated with the use of consisting photographs (e.g. ridge gourd curry was not illustrated separately as its portion sizes can be estimated using the photographs of snake gourd curry). In addition to the food items in the FFQ, a few food items that were revealed in the data collection phase of developing the FFQ, but have not been included in the FFQ as they did not contribute for 90% of the variance of total energy intake of carbohydrates, protein, total fat and dietary fiber, have also been included in the atlas. Certain western food items identified by consulting several nutrition experts were also included, due to increasing consumption of these foods by the urban population in Sri Lanka, which may be important for the future use of the atlas.

The main objective of this food atlas is to identify food portion sizes as well as food varieties. Therefore, selected food items were regrouped into three categories on the basis of their particular use.

1. Serial photographs: different portion sizes of a specific food item are illustrated, e.g. eight portions ranging from 100 g to 800 g of white rice.
2. Guide photographs: different food items were illustrated as they existed in the market.

- Different brands of a particular food item e.g. different brands of fresh milk bottles available in the market.
- Different types of food items belong to a certain food category, e.g. different types of Sri Lankan traditional sweets.

3. Range photographs: different sizes/shapes/varieties of a specific food item as it is available in the market, were illustrated, e.g. different sizes and varieties of mango available in the market.

The selected foods were divided into eight categories: 1) cereals or equivalents; 2) pulses; 3) vegetable dishes; 4) fish/meat or equivalents 5) fruits; 6) milk and dairy products; 7) desserts and sweets and 8) miscellaneous. Most of the food items in categories 1–4 were selected to be presented as serial photographs (3, 4, 6 or 8 portions as appropriate) in order to cover a range of consumption sizes from the smallest to the largest. Exemptions were made in presenting a few items, e.g. bread, pizza, Sri Lankan traditional sweets, which were illustrated as guide photographs to show the different types and sizes available for purchasing. It was decided to conduct a market survey to find different brands with different container sizes of milk, dairy products and ready to serve drinks, and different types of sweets, e.g. Sri Lankan traditional sweets, to represent the existing variation of food products and to illustrate as guide photographs. With reference to fruits, it was decided to represent large fruits, e.g. papaya, melon, in pieces of increasing sizes using serial photographs, and small fruits with certain size variations, e.g. mango, avocado, in a single photograph (Range Photographs) that depicts a range of disparate sizes.

### Determining portion sizes

#### As a series of photographs of increasing portion size

In Sri Lanka, rice is consumed as the staple food, and usually coconut shell spoons are utilized as the main household measure for serving rice. A medium sized coconut shell spoon can contain approximately100g. Previous studies have reported that an average Sri Lankan adult consumes approximately 400 g of boiled rice and, 200 g and 600 g are the 25th and 75th percentile, respectively [26]. Therefore, the portions for rice as well as other food items which are consumed as alternative staple foods are shown in increments of 100 g, as 8 portions from 100 g to 800 g. For the food items that are consumed in bulk mostly for breakfast, e.g. green gram, chick peas, cow pea, but in lower amounts than rice, half of a medium size coconut spoon (50 g in average) was used as the fixed increment for portion series.

Rice is served with curries made of vegetables and meat or equivalents, and the usual household measure is the table spoon, which can contain an average of 15 g. Hence, the range of portion sizes for most curries was determined with the fixed increment of 15 g used for each increasing portion. However, the accompanying dishes that are of light weight, e.g. dark green leafy salads, or usually consumed in small quantities, e.g. dry fish, were measured in 7.5 g increments (half of the fixed increment for other curries) for the series of portions. Dhal (lentils) curry, which is a common dish in most households, is prepared according to two main recipes; one that is made with a thick consistency and another which is watery in texture. Portion size series for thick dhal curry was determined as in the other common curries, but for the watery dhal curry, increment was taken as twice of the other (30 g), as it is consumed in higher amounts. For the above foods, six or eight photographs of increasing portion sizes were used as appropriate to represent the range of portion sizes.

A medium sized scoop was occupied in measuring dairy products, e.g. ice cream, curd, and the portion sizes were determined in scoop wise. Four different serving sizes were attributed to each dessert item, to cover up consumption variation. To determine portion sizes of traditional foods, e.g. roti, pittu, milk rice, ten housewives of local families were consulted. Some traditional foods, e.g. string hoppers, were purchased from local restaurants to represent the range of sizes available. Since there are no standard cups or glasses for the measurement of drinks, e.g. milk, fruit juice, the volume of an average cup (200 mL) was taken as the median portion and 100 mL and 300 mL were taken as the lower portion and higher portion, respectively. In cases where the foods were deemed to be purchased rather than prepared at home, e.g. breads, pizza, representative food samples were obtained from typical sources and the weights were recorded.

#### As a picture with a range of sizes of a particular food

Food items that differ in portion size along a range from very small to very large and foods that are irregular in shape or size, were presented using a single photograph which depicted all the portion sizes available in the local market. The market survey was conducted from January to May, 2016, at Colombo, Sri Lanka and all the sizes or shapes found during the survey were depicted in the range or guide photographs, e.g. ten mangoes have been illustrated in the range photographs to represent the different sizes and varieties of mangoes available in the local market. Two to four samples of the same item were purchased and one of those which the study team agreed as the most representative, was illustrated in the atlas, e.g. string hoppers of five varying sizes were purchase and three sizes of those, that could be categorized as small, medium and large, were selected to be depicted in the atlas. Moreover, the standard measuring unit (equivalent to 10 ml of absolute alcohol) were used to present liquor in quantities such as a beer mug, a wine glass and a shot glass.

### Food preparing or purchasing

Sri Lanka’s multi-ethnic culture has resulted in the consumption of a variety of mixed dishes; however, the recipes vary not only among different ethnic groups, but also from household to household. Therefore, steps in a standard recipe book [27] that were confirmed by seven housewives and nutritionists were followed when preparing local food items. Conversely, a few recipes had to be modified for generalization in order to represent the wide distribution of consumers, e.g. dhal curry. Most dishes were cooked in household kitchens, by pre-selected housewives. They were provided with guidance on how to prepare the food, e.g. the method of cutting a certain vegetable, how to cook the food, e.g. boil in coconut milk or temper with oil, and the approximate weight of the food item to prepare. A timetable was provided on indicating which foods were to be prepared on each day, while all the necessary raw material was supplied on the previous day. Three samples were prepared from each dish, and the most suitable one to be pictured was selected by the study team.

Some dishes were purchased from recognized local restaurants, e.g. fried rice, kottu, so as to reflect the food commonly consumed by Sri Lankans. Certain western food items were bought from popular international franchises, e.g. pizza from the Pizza Hut (https://www.pizzahut.lk) and French fries from the Kentucky Fried Chicken (KFC) (http://www.cargillsceylon.com/OurBusinesses/KFC.aspx). It was also decided to buy popular brands of dry rations, e.g. corn flakes, oats, and certain other food items of varying pack sizes, e.g. ice cream, milk, from leading supermarkets in Sri Lanka: Cargills Food City (http://www.cargillsceylon.com), Keells Super (www.keellssuper.com), Arpico Super Center (https://arpicosupercentre.com). Moreover, because it is essential to include all the common types of fruits, the project was extended to seven months in order to cover seasonal fruits.

### Food weighing

Each portion size or each food item was weighed by the research team on an electronic kitchen scale (Tanita KD 320, Japan) which had a maximum weighing precision of 0.1 g and a load capacity of 15 kg. Portions of liquid food items were measured using a standard measuring cylinder. The measured weights/volumes with the particular photo numbers were recorded and later entered in a data base.

### Food portraying

White color crockery was used to present most of the food items in the series of photographs of increasing portion sizes to create a sharp visual contrast. However, when the food item itself was white in color, a green colour banana leaf was placed on the crockery to distinguish the food item properly. For the food items in a range of sizes, only a blue colored board was used so that graphic artists could easily replace the background of the picture with another.

Since the size of Sri Lankan dishware varies significantly, a standard sized set of crockery with unique codes was bought from a reputed crockery manufacturer (Dankotuwa Porcelain, Sri Lanka). The weighed portions were presented on/in a 27-cm dinner plate (code: 0520), a 21 cm salad plate (code: 3611), or a 16.5 cm bowl (5 cm depth) (code:0307) as appropriate. For presenting dessert items and liquids, a dessert cup (code: 1,015,238) and glasses (codes:0842 and 5745) were used respectively. Majority of the Sri Lankans are in the habit of consuming foods by hand, so spoons and forks are not much in use. But table spoon was found to be a cutlery that is available in more or less similar sizes in all households. Therefore, a standard table spoon (code: 455) manufactured by a reputed company (Lanlo, Sri Lanka) was used as the standard reference object in all photographs to improve the respondent’s perception of the size of the food item or the plate/bowl on which the food portions were portrayed. Nevertheless, to avoid any misjudgments, a life size photograph of a dinner plate with the table spoon was printed at the end of the atlas (A3 sized page) as an aid to identifying the portion sizes depicted in the atlas.

### Photographing

All the selected food items were filmed by a professional photographer. To produce high quality and comprehensible digital images, a standard camera set up recommended for food photography was employed [28]. The serial photographs were taken using two cameras: one fixed above, and the other placed on the side of the food item. The former which captured aerial views, was placed at a distance of 85 cm (measured from the focal point to the film plate) and was set at an angle of 5° from the vertical plane to eliminate reflections of the camera on the images. The second camera, placed on the left side, capture dangled views at a distance of 90 cm (measured from the focal point to the film plate) and is set at an angle of 45° above the horizontal, which is considered to represent the view of a person of average height, sitting at a table, looking at a plate of food on the table in front of him. The liquid portions in a glass, e.g. milk, fruit juice, herbal gruel, were photographed at an angle of 90° to get life-size images. The range and guide photographs were taken free hand so that all the items could be captured in the best way possible.

A Nikon d7100 (Japan), with 18 -140 mm lenses (Nikon, Japan), camera was used for the development of the atlas. It was a 24.1 M pixel DSLR camera that used 1/25 speed, aperture f11, ISO 100, and a colour checker to optimize the colour result. The two cameras were connected to two laptops HP 1000 (China). Pictures were reviewed using the software program digicam control 2.0.0.0 (Open Source Project) to ensure the food and setting were consistent in each picture. Lighting was provided by two Nicephoto NI200 (Shenzhen Nice Photographic Equipment Co. Ltd., China) at a 45° angle, which was softened using two light soften material for each. External light was restricted using white clothing accordance with the set up recommended by Islam et al. [28] and internal lighting was arranged in order to improve the clarity of the photos without altering the original color of the food item.

## Results

### Foods selected

A total of 125 foods commonly consumed by the Sri Lankan adults were selected to be depicted in the atlas. The selection of food items was based on 3 sources, the local survey conducted for the development of a FFQ for Sri Lankan adults (*n* = 64 foods), market survey (*n* = 34 foods) and interviews with nutrition experts (*n* = 27 foods) (Table 1). The food items were sequenced as the most commonly consumed items appear first.

**Table 1:** Number of foods selected from different sources

Source	Serial	Range	Guide	Total
Local Survey	63	1	0	64
Market Survey	0	8	26	34
Interviews	25	2	0	27
Total	88	11	26	125

### Presentation of foods

**Fig. 1:**
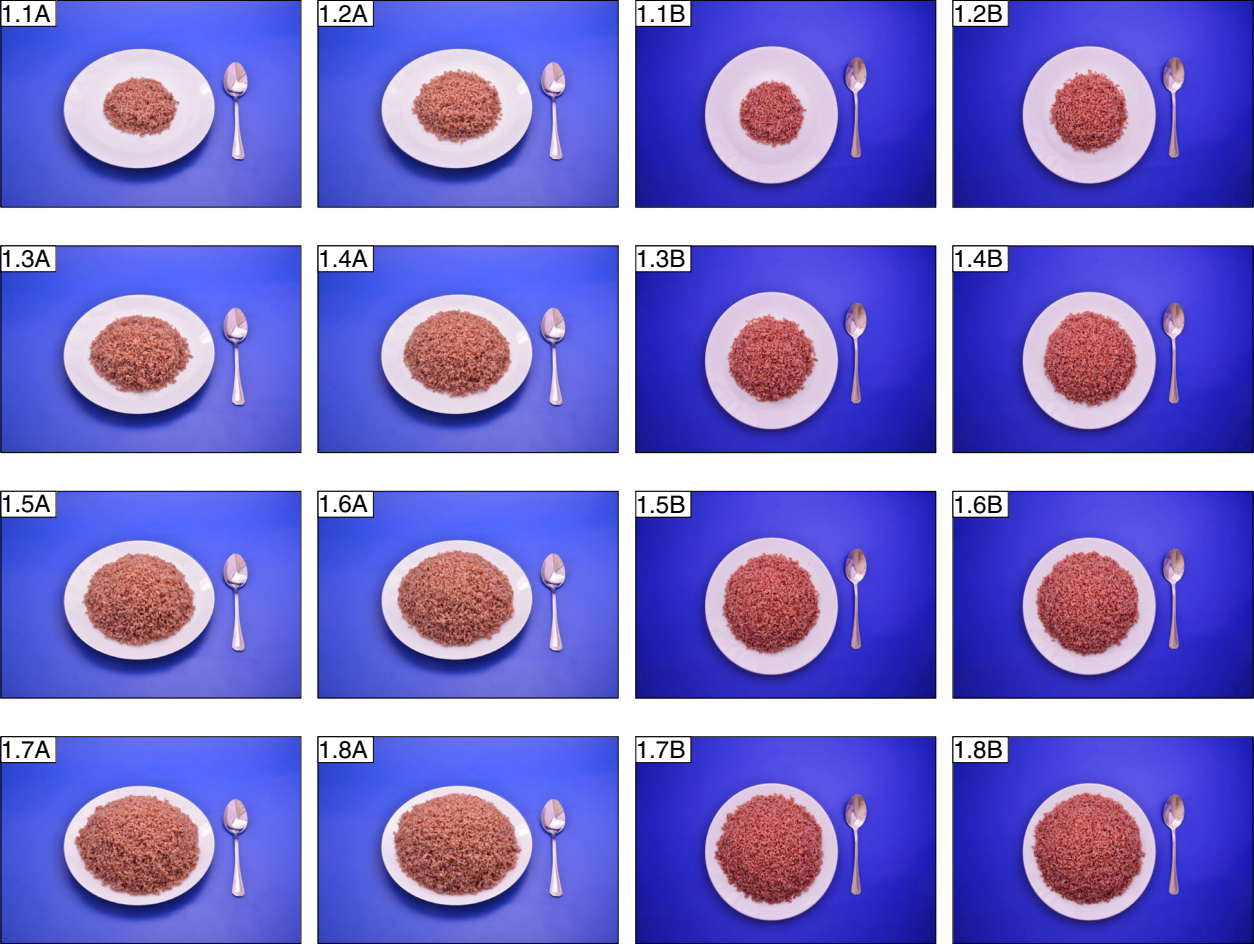
**a** Series of 8 photographs representing angled view (45°) of portion sizes of cooked red rice. Photographs are shown in increments of 100 g, as 8 portions from 100 g to 800 g, in the ascending order. **b** Series of 8 photographs representing aerial view (5°) of portion sizes of cooked red rice. Photographs are shown in increments of 100 g, as 8 portions from 100 g to 800 g, in the ascending order

Three types of photographs have been used to illustrate the food items: 1) serial photographs of increasing sizes of eight portions (*n* = 64 foods), six portions (*n* = 7 foods), four portions (*n* = 12 foods), and three portions (*n* = 5 foods), 2) range photographs depicting a range of sizes or varieties of a particular food in a single photograph (*n* = 11), and 3) guide photographs which represent the brands/types of a certain food item/category available in the market in a single photograph (*n* = 26) (Table 2). Additional file 1 provides the details on the type of photograph used to portray each food item. The serial photographs were presented as two successions of two angles portrayed in consecutive pages; series of photographs of angled view (45°) and series of photographs of aerial view (5°); for clear identification of height and area of the portion sizes (Fig. 1a and b). Two additional photographs depicting the selection of household measures (plates and cups, spoons) and a separate life sized photograph of the dinner plate with the table spoon used in presenting the food items, were also included to facilitate easy description of portions.

**Table 2:** Presentation of foods according to different photograph categories

Category	Representation	No. of food items	Total
Serial Photographs	Series of 8 portions	64	88
	Series of 6 portions	7	
	Series of 4 portions	12	
	Series of 3 portions	5	
Range Photographs	Different sizes/shapes/varieties	11	11
Guide Photographs	Different brands of a specific food	10	26
	Different types in a specific food group	16	
Grand Total			125

### Portion sizes

The majority of the portion sizes for serial photographs (*n* = 63) were determined on the basis of the local survey on dietary habits of Sri Lankan adults [16]. When data on the distribution of portion sizes of a particular food item were not available, portion weights were determined based on the amounts suggested by nutrition experts, which were later confirmed by local housewives (*n* = 27 foods). Of the eleven food items depicted as range photographs, eight illustrated sizes and varieties available in the market. Other sources (local survey [*n* = 1] and consulting experts [*n* = 2]) contributed for three food items. All the guide photographs (*n* = 26) reflect what is commonly available in the market (Table 1).

### Size of photographs

**Fig. 2:**
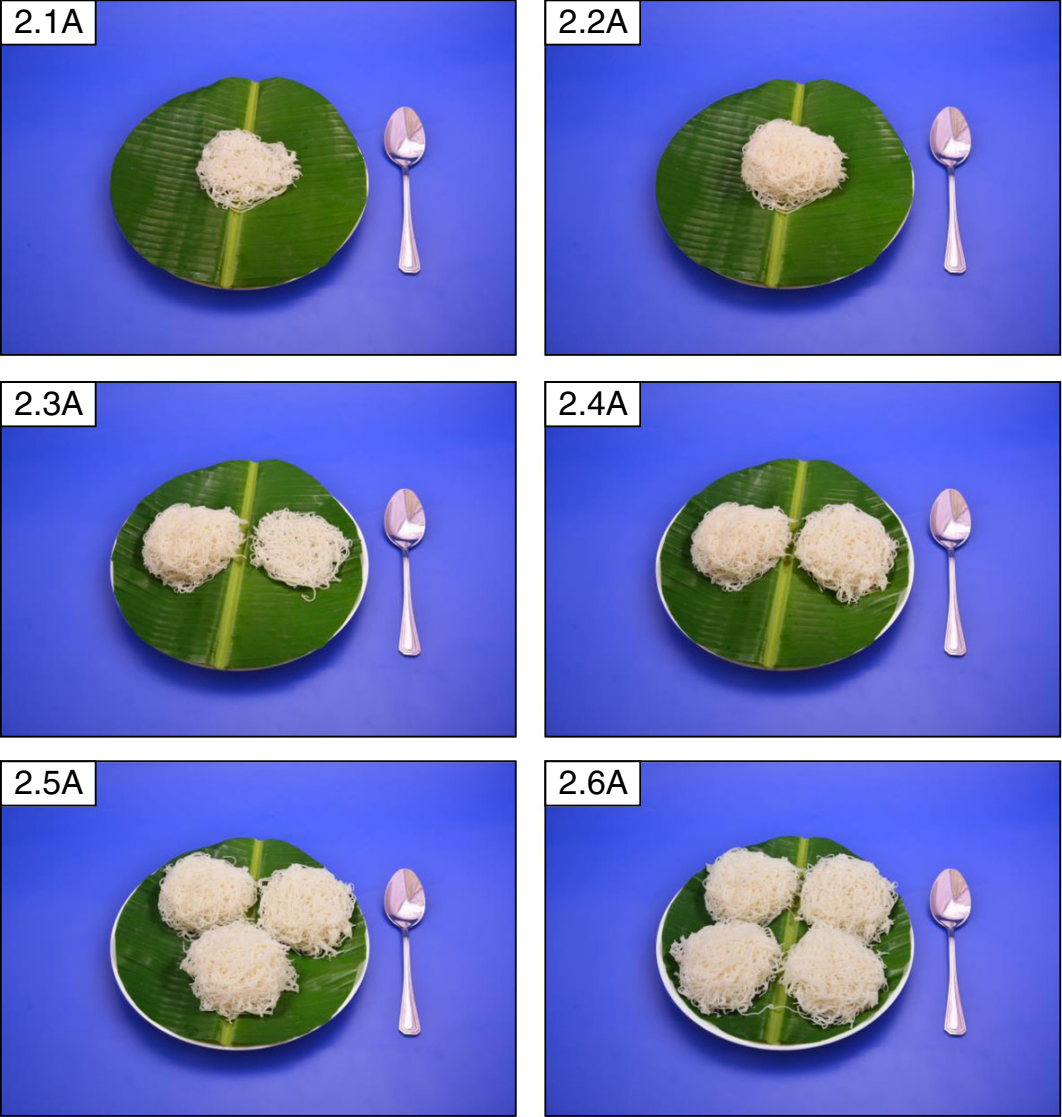
Series of 6 photographs representing angled view (45°) of portion sizes of string hoppers. The weights of the portions are 34 g, 91 g, 128 g, 186 g, 280 g, 372 g, 464 g and 550 g, in the ascending order

**Fig. 3:**
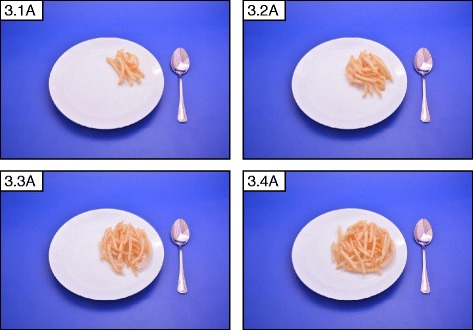
Series of 4 photographs representing angled view (45°) of portion sizes of French fries. The weights of the portions are 25 g, 50 g, 75 g and 100 g, in the ascending order

**Fig. 4:**
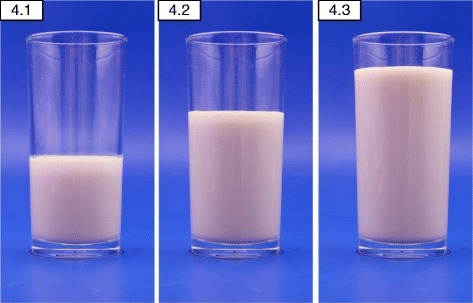
Series of 3 photographs representing front view of portion sizes of milk. The volumes of the portions are 100 mL, 200 mL and 300 mL, in the ascending order

Eight A7 sized (75*60 mm) photographs per food were chosen which allowed 8 photographs to be clearly displayed together on one A4 page facilitating easy comparison. The portions were arranged in ascending order with the smallest portion at the top left corner. Exemptions were made in the number of portions (6, 4 or 3 portions as required) when there was less variation in the amount consumed. Figures 1a, b, 2, 3 and 4 show examples for photographs with portions 8, 6, 4 and 3, respectively.

**Fig. 5:**
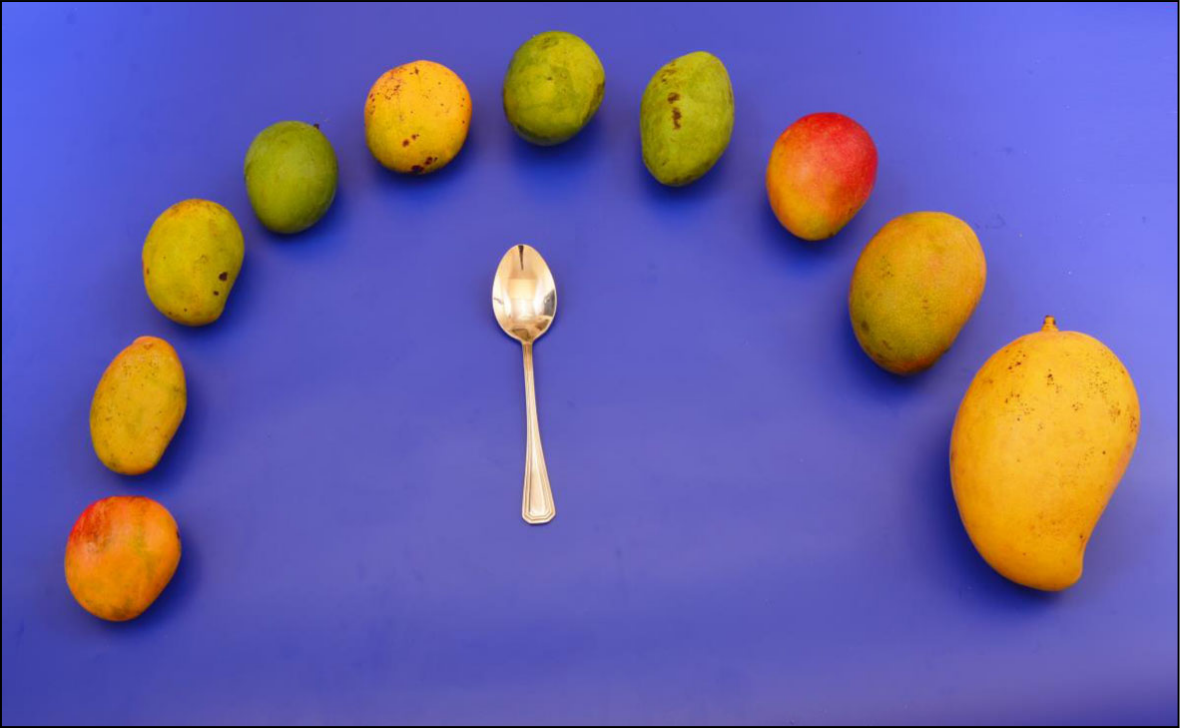
Range photograph representing different sizes and varieties of mango available in Sri Lankan market

**Fig. 6:**
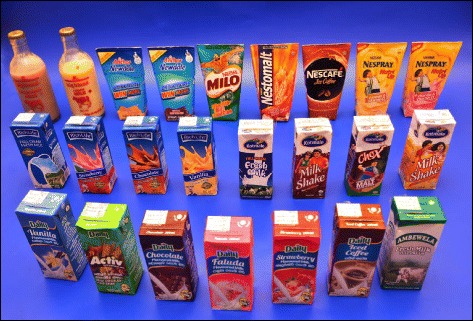
Guide photograph representing variety of ready to serve milk products in Sri Lankan market

**Fig. 7:**
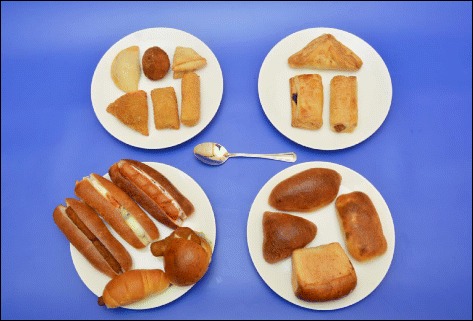
Guide photograph representing variety of buns and pasties available in Sri Lankan market

The range photographs depicted different sizes/shapes/varieties of a specific food item available in the market on one A4 page (Fig. 5: Different sizes and varieties of mango available in the market). A selection of food items of different varieties as they exist in the market were illustrated in guide photographs, which were further categorized into two groups:1) different brands of a particular food item of a specific weight (Fig. 6: different brands of ready to serve milk products available in the market); 2) different types of food items belonging to a certain food category (Fig. 7: different types of buns and pasties available in Sri Lankan market).

**Fig. 8:**
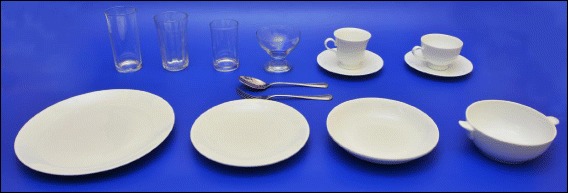
Selected crockery and cutlery commonly used by Sri Lankans

Additional guide photographs of cutlery and crockery have been used to enhance the perception of the portion sizes depicted in the atlas. Fig. 8 represents the selected crockery and cutlery commonly used in Sri Lanka.

## Discussion

Different types of visual aids, e.g. actual foods and containers, food replicas or other three-dimensional models, household utensils, published standard portion sizes, drawings and food photographs) are often used to assist respondents in describing portion sizes which may help improve the accuracy of food portion quantification [29]. However, portion estimation using photographs seems to be the most practical method, as photographs can be made of any required portions of any food, and can be conveniently carried by interviewers [30]. Nevertheless, a previous study has confirmed that presenting aids in the form of photographs, other two-dimensional representations, or three-dimensional models appear to yield similar results [11].

Nelson et al. recognized three main elements in the process of assessing food portion sizes using photographs; perception, conceptualization and memory [31]. Perception is the subject’s ability to relate a quantity of food which is present in reality to an amount illustrated in a photograph. Conceptualization is the subject’s ability to develop a mental picture of a food portion not actually present and to relate it to a photograph. Memory is the subject’s ability to accurately recall the quantity of food eaten and will influence the accuracy of conceptualization [32]. These three elements are affected by the number of different food portion sizes, their placement in the food atlas, the dimensions of each photograph, the camera angle by which each of them was captured [18], the age of the subject [33], and the use or non-use of household objects during the test [34].

The number and sizes of photographs employed to describe food portions may vary among studies [35]. On occasion, the subjects may be asked to describe their usual portion as a fraction, multiple or a percentage from an average portion size shown in a single life-sized photograph. Although it seems life size photographs may provide more accurate results, according to the findings of research carried out to determine the most appropriate format for the photographs, larger prints have not produced a significant increase in accuracy compared to smaller prints. Besides, larger prints are expensive to develop and impractical to use [31]. A visual analogue scale (VAS) is a measurement technique used to estimate the approximate portion of an individual, from a series of images depicting a range of portion sizes [31]. In most food frequency questionnaires, three portion sizes (small, medium, large) representing the range of portions consumed by the target population, have been used for estimating portions [18]. It has been found that using four or more photographs per food item may strengthen reporting accuracy [36]. Moreover, when the portions are in odd numbers subjects tend to select the middle photograph. Thus an even number of photographs(four, six or eight) is better [31]. However, fewer photographs (e.g. four vs. eight) result in some loss of precision [22]. Eight A7 (6 × 8 cm) sized photographs in a single A4 page which have been adopted to illustrate series of portion sizes in atlases of developed countries (UK and UAE), has also been used in our atlas. This method presents considerable variation in the portions and allows subjects to compare between portions easily [25].

The portion sizes which are depicted in an atlas must be identified using population-based data [22]. Portion sizes ranging in weight from the 5th to 95th centile of the distribution of portion sizes observed in the Dietary and Nutritional Survey of British Adults by Gregory et al., 1990, has been used for determining the eight portions in the UK atlas [31]. The UAE atlas has replicated the portion sizes of the UK atlas, not only for foods identified as being commonly consumed in both the UAE and UK, but also for some foods deemed to be equivalent to foods in the UK atlas [25]. The portion sizes for Sri Lankan Food atlas were mainly based on a nationally representative survey of which dietary intakes of Sri Lankan men and women were assessed using a small sample of adults [16]. Although the data did not result in a perfect distribution due to limited sample size, it was the only available data representing dietary information of Sri Lankan adults.

This is the first comprehensive food atlas designed for Sri Lankan adults which will be used in future food consumption surveys of Sri Lankans living in the country as well as abroad. This may also assist in portion size estimations in other south Asian countries where similar food items are consumed (e.g. rice, vegetable curries). The atlas will serve as a valuable tool in identifying the current intake of the subject by asking the individual to select the portion size from a series of portions. As the nutrition compositions (energy, carbohydrate, protein and fat content) of respective food items are given, current energy and nutrient intake of the individual can be easily calculated. Since this is closely related with the food items of a validated FFQ, this can be used along with the FFQ to obtain a better estimation of individual energy and nutrient intake. Moreover, for estimating the intake of food items that have not been presented in the atlas, portion sizes of food items from the atlas with similar consumption patterns (i.e. considered to be equivalent in terms of food type, portion size, composition and physical attributes) can be employed. For example, snake gourd curry which is included in the atlas is similar to ridge gourd curry, which was not selected for inclusion in the atlas and therefore the portion weights for snake gourd curry in the atlas can be used to estimate the intake of ridge gourd curry. Owing to the extensive illustration of most of the common food items that are available in the local market, this atlas is a very useful tool to introduce and differentiate commercially available food items (e.g. differentiate non-fat and low-fat milk products). There are a several ethnic groups in Sri Lanka and they have food items which are unique to their culture, especially sweets. As the atlas consists of common food items from the different ethnic groups, it can be used nationally. Although the cost of black and white reproduction is cheaper, and previous research manifests that there was no difference between coloured or black and white photographs relating to errors in the estimation of portion size, the food atlas was printed using coloured photographs, not only because it illustrates the foods better, but also because it can better attract the subject’s attention, especially in a long interview [22].

Regardless of the number of advantages, there are a few limitations in using photographs as visual aids to estimate food portion sizes. A study has found that the errors which are frequently occurring when estimating portion sizes using photographs, were generally small (<10%) to moderate (10-25%) [11]. Nelson et al. have revealed the flat slope phenomenon and a regression to mean effect; small portion sizes are overestimated, large portion sizes are underestimated and medium portion sizes usually estimated comparatively well [32]. In a large cohort study on dietary habits (of 23 different dishes), the volunteers overestimated the portion size by more than 20% for six foods and underestimated the portion size by more than 20% for four foods [37]. The investigators revealed a correlation between overestimation of portion size by those who ate smaller portions and underestimation by those who ate larger portions [37]. It has conclusively been shown that under- or overestimations occurred most likely with respect to the certain visual characteristics of the foods or how the food was presented. For example, a given amount of food can be portrayed in the corresponding photograph in pieces which were either smaller and more numerous or larger and less numerous than in reality [11]. For some foods the way it is presented may become a limiting factor when determining their portion (e.g. foods that are not usually eaten on a plate; like nuts; were photographed in a plate but many people would consume it in handfuls) [24]. Another limitation is that an atlas in which the portion sizes were derived from adults’ data cannot be used in estimating diet intake of children. A study in UK found that children gave greater overestimations of portion sizes [30] and a possible explanation for this might be that the children were used to consuming smaller portion sizes than the smallest portion size photographed in the food atlas [31]. This view is supported by the argument that the children’s immature cognitive skills render estimating portion sizes more difficult for them compared to adults [38]. Another survey highlights the need for age-appropriate food photographs for improving the quality of dietary intake data collected from children [39]. In addition to those general limitations, the given calorie values corresponding to portions of some dishes may differ because of the varying recipes around the country. Furthermore, as Sri Lanka is undergoing nutrition transition, the foods depicted in the atlas will need to be updated according to the people’s dynamic food habits and market composition.

## Conclusion

Obtaining reliable food portion size estimations has been found to be a difficult task unless the subjects are assisted with visual aids. Providing aids in the form of photographs which represent a range of portion sizes seems to be the most practical method. A food atlas is a set of photograph series that depict different amounts of a particular food, usually bound together in a single volume. This is the first food atlas which has been developed for use in dietary surveys in Sri Lanka. This may also assist in portion size estimations in other South Asian countries where similar food items are consumed.

## Additional file

Additional file 1: Details on types of photographs employed to depict the food items. (XLSX 21 kb)

## Abbreviations

24HR: 24-Hour Dietary Recall
DH: Dietary History
DR: Dietary Record
FFQ: Frequency Questionnaires
NCDs: Non-Communicable Diseases
UAE: United Arab Emirates
UK: United Kingdom
USA: United States of America
VAS: Visual Analog Scale
